# Muscle thickness correlates to muscle cross‐sectional area in the assessment of strength training‐induced hypertrophy

**DOI:** 10.1111/sms.12961

**Published:** 2017-09-21

**Authors:** M. V. Franchi, S. Longo, J. Mallinson, J. I. Quinlan, T. Taylor, P. L. Greenhaff, M. V. Narici

**Affiliations:** ^1^ MRC‐ARUK Centre for Musculoskeletal Ageing Research University of Nottingham Nottingham UK; ^2^ Laboratory for Muscle Plasticity Department of Orthopaedics Balgrist University Hospital University of Zurich Zurich Switzerland; ^3^ Department of Biomedical Sciences for Health Università degli studi di Milano Milan Italy

**Keywords:** anatomical cross‐sectional area, magnetic resonance imaging, ultrasound, volume

## Abstract

Muscle thickness (MT) measured by ultrasound has been used to estimate cross‐sectional area (measured by CT and MRI) at a single time point. We tested whether MT could be used as a valid marker of MRI determined muscle anatomical cross‐sectional area (ACSA) and volume changes following resistance training (RT). Nine healthy, young, male volunteers (24 ± 2 y.o., BMI 24.1 ± 2.8 kg/m^2^) had *vastus lateralis* (VL) muscle volume (VOL) and ACSA_mid_ (at 50% of femur length, FL) assessed by MRI, and VL MT measured by ultrasound at 50% FL. Measurements were taken at baseline and after 12 weeks of isokinetic RT. Differences between baseline and post‐training were assessed by Student's paired *t* test. The relationships between MRI and ultrasound measurements were tested by Pearson's correlation. After RT, MT increased by 7.5 ± 6.1% (*P* < .001), ACSA_mid_ by 5.2 ± 5% (*P* < .001), and VOL by 5.0 ± 6.9% (*P* < .05) (values: means ± SD). Positive correlations were found, at baseline and 12 weeks, between MT and ACSA_mid_ (*r *= .82, *P* < .001 and *r *= .73, *P* < .001, respectively), and between MT and VOL (*r *= .76, *P* < .001 and *r *= .73, *P* < .001, respectively). The % change in MT with training was correlated with % change in ACSA_mid_ (*r *= .69, *P *< .01), but not % change in VOL (*r *= .33, *P *> .05). These data support evidence that MT is a reliable index of muscle ACSA_mid_ and VOL at a single time point. MT changes following RT are associated with parallel changes in muscle ACSA_mid_ but not with the changes in VOL, highlighting the impact of RT on regional hypertrophy.

## 1 INTRODUCTION

Skeletal muscle is the largest adipose tissue‐free mass in humans, constituting a substantial portion of the whole‐body mass, and it is crucial for locomotion and metabolic health. Over the last four decades, the quantification of skeletal muscle mass has been revolutionized by the introduction of imaging techniques such as computer tomography (CT), magnetic resonance imaging (MRI) and dual‐energy X‐ray absorptiometry (DXA), which facilitate the accurate quantification of whole‐body and regional muscle masses.^1^ These techniques have been used in a variety of settings, yet can be expensive, often inaccessible and, in the case of CT and DXA, involve ionizing radiation.

MRI is regarded as the gold standard for clinical and research imaging of skeletal muscle, allowing investigators to accurately assess muscle mass at an individual time point and its changes over time.^2^, ^3^ However, besides its accuracy, estimation of whole‐body muscle mass is not as cheap and accessible as with other techniques. DXA, for example, can provide estimates of regional and total lean masses at a lower cost than MRI and involves minimal radiation exposure compared to CT.^1^ Nonetheless, repeated DXA scanning in longitudinal studies does raise ethical concerns because of the stochastic risk posed by repeated radiation exposure.

Over the last 20 years, the use of ultrasound has been advocated as a potentially reliable tool for the quantification of skeletal muscle mass in young and older healthy volunteers^4^, ^5^, ^6^ and in clinical populations, such as intensive care patients.^7^, ^8^, ^9^, ^10^ Previous studies report a positive relationship between muscle thickness (MT) and lean mass (measured by DXA),^11^, ^12^ MT and anatomical cross‐sectional area (ACSA, measured by MRI),^13^ and between MT and muscle volume (VOL, measured by MRI)^14^, ^15^, ^16^ at a single time point. However, as far as we are aware, no study has reported the utility of MT measurements for detecting changes in muscle size or volume induced by resistance exercise training (RT).

Hence, the aim of this study was to examine whether muscle thickness measurements from ultrasound could be used to accurately estimate changes in muscle size and volume (assessed with MRI) following a RT protocol. It was hypothesized that *vastus lateralis* (VL) MT assessed at a single time point using ultrasound would be positively correlated with quantification of VL ACSA and VOL using MRI at the same time point. The second hypothesis was that the RT‐induced change in VL MT would be positively correlated to changes in VL ACSA and VOL.

## 2 METHODS

### 2.1 Participant characteristics and study design

Nine recreationally active, young, healthy males (age* *= 24 ± 2 years, BMI* *= 24.1 ± 2.8 kg/m^2^) volunteered for this study. Each participant underwent 12 weeks of unilateral RT performing maximal knee extensions on a Cybex® isokinetic dynamometer. One leg was trained concentrically (5 sets × 30 repetitions at 90 deg/s), whereas the contralateral limb was trained both concentrically (2 sets × 30 repetitions at 90 deg/s) and eccentrically (3 sets × 30 repetitions at 90 deg/s). This protocol was used to vary the training stimulus between legs and thereby potentially induce different increases in muscle mass between legs. Both legs performed knee extensions throughout the whole range of motion. Training frequency was 3 times per week. The choice of leg exercise combination was randomized. VL ACSA and VOL were measured at baseline and at 12 weeks using MRI (GE, 3T 750 Discovery, Chalfont Saint Giles UK). MT was also measured by ultrasonography at the same time points. Thus, a total of 18 (ie, 9 volunteers, both legs tested and trained differently) values of VL MT, ACSA and VOL were obtained per time point. The study was approved by the University of Nottingham Medical School Ethics Committee, in accordance with the Declaration of Helsinki, and informed consent was obtained from all participants.

### 2.2 VOL and ACSA assessments

**Figure 1:**
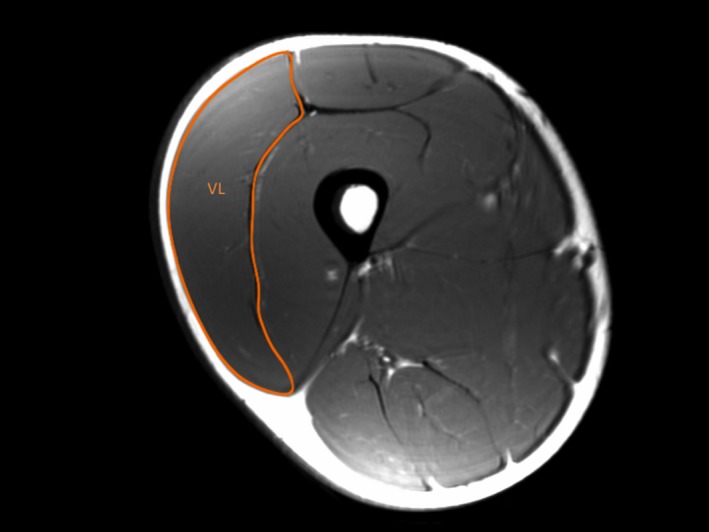
Magnetic resonance image scan of the right thigh at 50% of femur length from a representative subject. The contours that comprise the *vastus lateralis* (VL) anatomical cross‐sectional area are shown

Axial plane scans of each thigh were obtained using an MRI scanner (GE, 3T 750 Discovery). A T1‐weighted Spin Echo protocol was used (repetition time 600 ms, echo time 15.2 ms, field of view 512 × 512 mm, slice thickness 10 mm, no gap between slices). Participants were asked to lie supine on the MRI bed for 20 minutes to allow body fluid shift stabilization. Thereafter, 38 axial plane scans along the entire length of the VL were collected. From these scans, the contours of the VL muscle of each MRI scan were digitized offline using the Osirix DICOM image analysis software (Pixmeo, Geneva, Switzerland) (Figure 1). When it was difficult to distinguish the contours of VL and vastus intermedius muscles (ie, usually close to the very proximal insertions), the remaining ACSA (n* *= 2‐3 circa) were estimated by fitting the other obtained values into a spline curve.^17^, ^18^ Subsequently, VL VOL was calculated as previously described^19^ using the following equation:

$${\text{Volume}}_{\mathrm{VL}}\left({\text{cm}}^{3}\right)=\sum_{\mathrm{ACSA}}\cdot \text{slice thickness},$$

where Σ_ACSA_ is the sum of contiguous ACSA, and slice thickness refers to the thickness of each individual MRI axial image with no gap between contiguous slices.

VL muscle ACSA was measured at 50% of femur length (ACSA_mid_), defined as the distance from the greater trochanter to the lateral border of the femoral condyle (which was previously measured from a single coronal image). To ensure that ultrasound measurements were performed at the same anatomical location, this reference point was marked on the skin using an indelible pen.

### 2.3 MT assessment

VL MT was assessed by the same investigator from images obtained in vivo at rest using B‐mode ultrasonography (Mylab 25; Esaote Biomedica, Genova, Italy), with a 50 mm, 7.5 MHz, linear‐array probe. MT has previously been assessed by placing the ultrasound probe transversally in relation to the limb and evaluated as the perpendicular distance between the skeletal muscle interfaces.^20^ Longitudinal ultrasound scans (ie, with the probe aligned with the fascicle plane) have also been used to detect changes in muscle size and growth as well as skeletal muscle architecture.^21^, ^22^, ^23^

**Figure 2:**
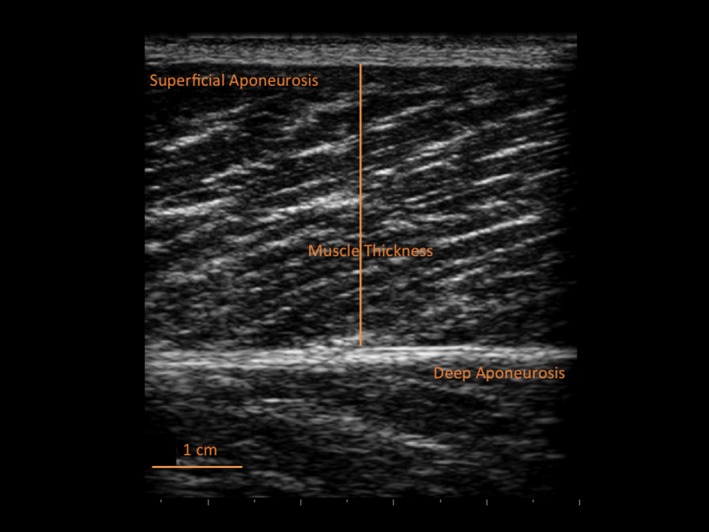
Ultrasound image of the *vastus lateralis* muscle (at 50% of femur length) from a representative subject with the muscle thickness measurement highlighted (solid line between aponeuroses)

In this study, resting ultrasound images were taken at 50% of femur length, applying the same reference point used for the MRI scanning. The participant was resting supine on an examination bed with the knee in full extension (ie, anatomical zero).^24^ The transducer was placed longitudinally to the thigh along the mid‐sagittal axis of the VL, and carefully aligned to the fascicle plane to clearly visualize fascicles on the ultrasound screen. The experienced operator was careful in applying as little pressure as possible when placing the probe on the skin. Three images were acquired and stored for offline analysis. VL MT was measured as the distance between superficial and deep aponeuroses, in the proximal, central, and distal portions of the acquired image^22^, ^23^ (Figure 2), using the image analysis software ImageJ 1.42q (National Institutes of Health, USA). The mean of the three measures was calculated for statistical analysis.

The reliability of this ultrasound technique has been previously validated by cadaver anatomical inspection.^25^ Moreover, previous studies assessed the reliability of in vivo measurements of fascicle length^26^ and pennation angle.^27^ In this study, the interday reliability of MT was also assessed. All subjects were tested on two different days before the start of the training period. Volunteers were tested at the same hour of the day, and a permanent marker was used to trace the ultrasound probe contours in order to ensure that MT was assessed at the same VL site on both days. All images were collected and digitally analyzed by the same operator.

### 2.4 Statistical analysis

All statistical analyses were conducted using the SPSS 23.0 software (SPSS® Inc., Chicago, IL, USA). Data are reported as mean ± SD. Normality of distribution was checked by the Shapiro‐Wilk's test. Differences between pre‐ and post‐training were statistically analyzed for muscle VOL, ACSA_mid,_ and MT values using paired Student's *t* test. Differences between legs at both time points were statistically analyzed using a two‐way repeated‐measures ANOVA. Correlations were tested using the Pearson's product moment correlation coefficient (*r*). The level of significance was set at *P* < .05.

The magnitude of the changes between baseline and 12 weeks was determined using effect size (ES) statistics with 90% confidence intervals (CI), or partial eta‐squared (η^2^) statistics when appropriate. ES was classified as trivial for ES values <0.20, small between 0.20 and 0.60, moderate between 0.61 and 1.20, large between 1.21 and 2.0, and very large when >2.0.^28^

The interday reliability of MT measurements was tested with the intraclass correlation coefficient (ICC, two‐way random, absolute agreement),^29^ with 95% CI, and the calculation of the relative standard error of measurement (SEM%). The minimum detectable change at 95% confidence as a percentage (MDC_95%_) was also determined.^30^ ICC values were considered as very high if >0.90, high if between 0.70 and 0.89, and moderate if between 0.50 and 0.69.^31^

## 3 RESULTS

### 3.1 MT reliability

Interday measurements of MT yielded an ICC of 0.99 (95% CI* *= 0.96‐0.99), with a SEM% of 1.65 and a MDC_95%_ of 4.6%.

### 3.2 Morphological adaptations

No differences were found between legs at both baseline and after training (*P *= .83). Following the RT protocol, all parameters were significantly different from baseline (Table 1). ACSA_mid_ increased by 5.2 ± 5%, (*P* < .001, ES* *= 1.05 ± 0.11, moderate), VOL by 5.0 ± 6.9%, (*P* < .05, ES* *= 0.69 ± 0.14, moderate), and MT by 7.5 ± 6.1%, (*P* < .001, ES* *= 1.28 ± 0.13, large). The observed mean changes in MT were greater than the requested MDC_95%_ (4.6%).

**Table 1:** *Vastus lateralis* anatomical cross‐sectional area measured at midpoint of femur length (ACSA_mid_) and total volume (VOL) measured by magnetic resonance imaging, and muscle thickness (MT) measured by ultrasound at the same site, before and after 12 wk of resistance exercise training

	Baseline	12 wk	*P*‐value	Effect size
ACSA_mid_ (cm^2^)	32.5 (5.4)	34.6 (4.6)	<.001	1.05
VOL (cm^3^)	668 (121)	695 (100)	<.05	0.69
MT (cm)	2.54 (0.4)	2.73 (0.34)	<.001	1.28

**Figure 3:**
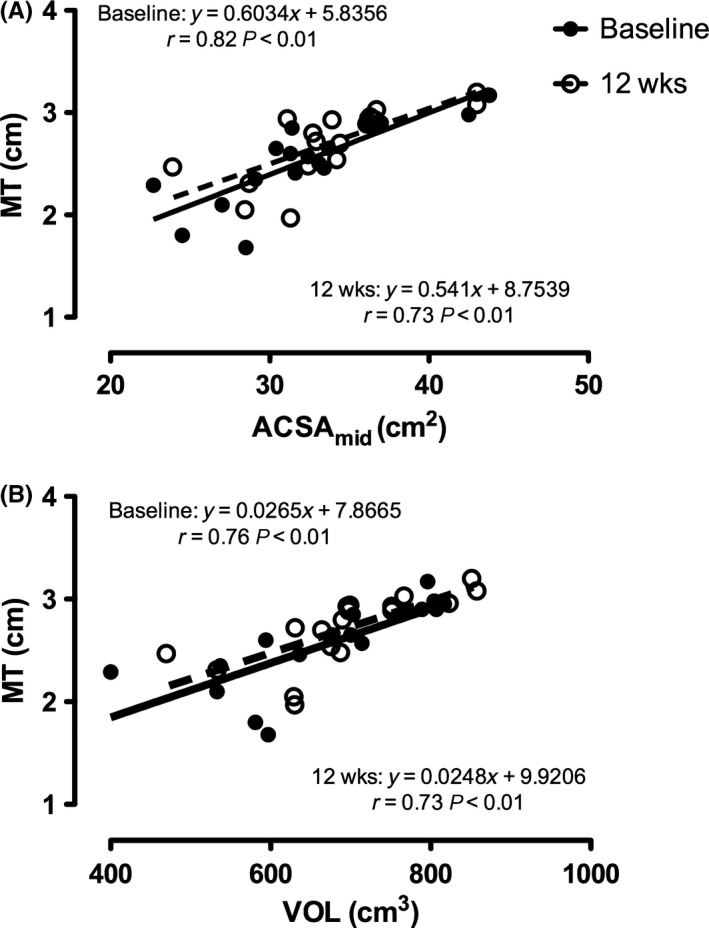
(A) Correlations between *vastus lateralis* cross‐sectional area measured at midpoint of femur length (ACSA_mid_) by magnetic resonance imaging and muscle thickness (MT) measured by ultrasound at the same site, before (filled circles, black line) and after 12 wk (empty circles, dashed line) of resistance training (RT). (B) Correlations between *vastus lateralis* whole volume (VOL) measured by magnetic resonance imaging and MT before and after 12 wk of RT. Participants N* *= 9. Data represent both legs for each participant (18 points)

When plotting ACSA_mid_ against MT (Figure 3A), significant positive correlations were found at baseline (*r *= .82 *P* < .001) and 12 weeks (*r *= .73 *P* < .001). Likewise, significant positive correlations were found between VOL and MT at both time points (baseline: *r *= .76, *P* < .001, very large; 12 weeks: *r *= .73, *P* < .001, very large; Figure 3B).

**Figure 4:**
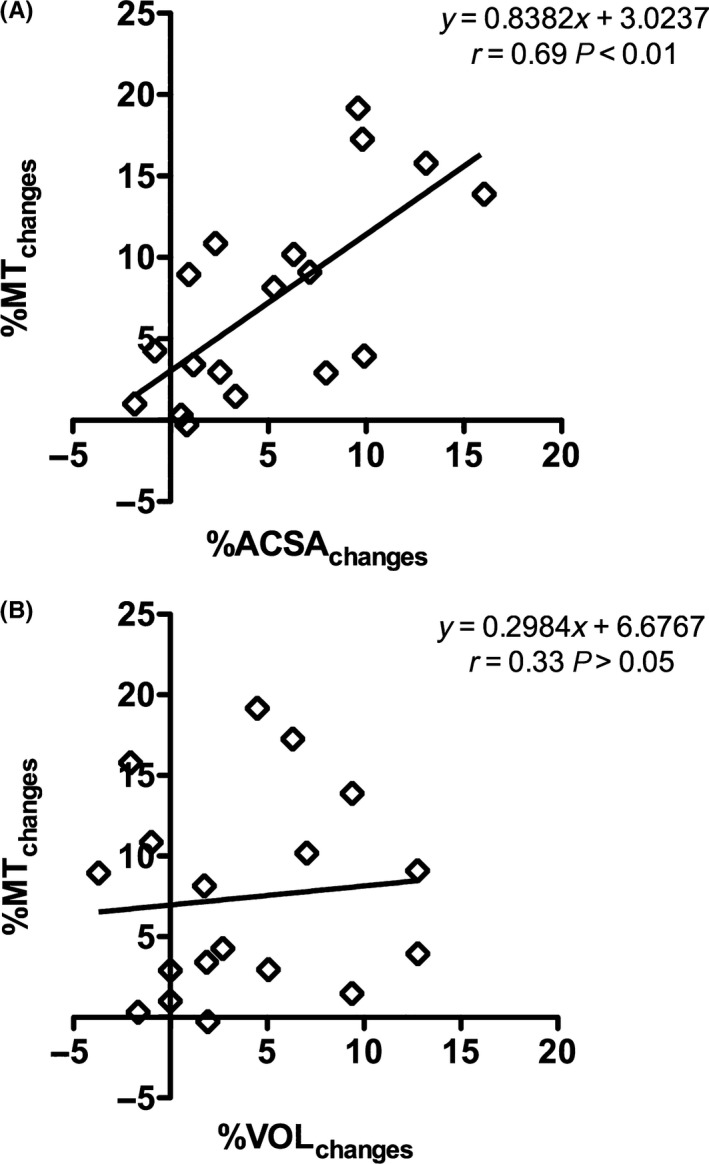
(A) Correlations between the percentage changes in *vastus lateralis* cross‐sectional area measured at midpoint of femur length (%ACSA_changes_) by magnetic resonance imaging and muscle thickness (%MT_changes_) measured by ultrasound at the same site induced by 12 wk of resistance training (RT). (B) Correlations between the percentage changes in *vastus lateralis* whole volume (%VOL_changes_) measured by magnetic resonance imaging and %MT_changes_ induced by 12 wk of RT. Participants N* *= 9. Data represent both legs for each participant (18 points)

A significant positive correlation was found between the percentage increase in VL ACSA_mid_ and percentage increase in MT (*r *= .69, *P *= .001 large; Figure 4A). However, no significant relationship was found between the percentage increases in VOL and MT (*r *= .33, *P *= .207, Figure 4B).

## 4 DISCUSSION

The present study demonstrated that VL MT measured mid‐thigh level using ultrasound before and after a knee extension training protocol was significantly correlated to ACSA_mid_ and VOL assessed by MRI at the same time points. However, when changes in muscle size were expressed as a percentage increase over the training period, only the increase in ACSA_mid_ significantly correlated with the increase in MT. These data support evidence that MT can be regarded as a readily available measure of muscle size that is related to skeletal muscle ACSA_mid_ and VOL when assessed at a single time point. Moreover, the results demonstrate that MT changes following RT are associated with parallel changes in muscle ACSA_mid_. However, the lack of association between relative changes in MT and relative changes in VOL highlights the impact of RT on regional hypertrophy.

Several studies have reported the measurement of MT in different scenarios to assess muscle hypertrophy,^22^, ^23^, ^32^, ^33^, ^34^, ^35^, ^36^ atrophy, and/or sarcopenia.^34^, ^37^, ^38^, ^39^, ^40^ Other studies have compared ultrasound MT measurement to muscle mass/volume assessed by either DXA or MRI.^6^, ^11^, ^13^, ^14^, ^16^, ^41^ However, to the best of the authors' knowledge, the present study is the first to investigate RT‐induced changes in MT and ACSA or VOL, respectively, assessed by ultrasound and MRI.

In light of the correlations between the changes in ACSA_mid_ and MT, it can be concluded that a single longitudinal ultrasound snapshot is sensitive enough to indicate the presence of VL muscle hypertrophy after a 12‐week RT program. However, a significant correlation between the percentage increase in MT and ACSA_mid_ is opposed to a non‐significant correlation between the changes in MT and VOL. This indicates that a single‐site ultrasound snapshot can detect changes in muscle size but also that these variations are not predictive of changes in muscle volume, which are affected by a heterogenous distribution of hypertrophy.^19^, ^42^, ^43^, ^44^ From a simple mathematical point of view, the three parameters (MT, ACSA, and VOL) would be expected to change proportionally only if the muscle had a perfectly regular geometrical shape (eg, ellipsoid); in fact, if assuming that the length and the width of a muscle are constant, the increases in muscle VOL should be reflected in a proportional increases in ACSA and MT.

However, the VL muscle does not seem to reflect these geometrical properties in response to knee extension training, as the present findings show different percentage changes among the three measurements. Therefore, as VL MT is canonically assessed at ~50% of the whole muscle length, the measured increase in muscle size at this site might not be representative of the changes occurring along the muscle belly, which reasonably reflect a regional distribution of hypertrophy.^19^, ^42^, ^43^, ^44^ Moreover, it should be noted that such relationships between MT, ACSA, and VOL could be specific to the type of training adopted in the present study (ie, knee extension). A previous investigation^44^ has reported regional hypertrophy in quadriceps muscles using a similar training protocol compared to this study, but it is a possibility that other typologies of RT that imply multijoints movements may elicit different regionally specific responses. However, similar regional hypertrophic responses have been reported for VL muscle when RT (concentric‐only vs eccentric‐only protocols) was performed using a leg press machine.^19^ Concentric‐only RT led to greater VL hypertrophy (in terms of relative increases in ACSA) in the middle of the muscle, and eccentric‐only RT presented more pronounced distal growth. In the present investigation, although both legs performed the same amount of repetitions, one leg was trained with an additional eccentric component. Thus, it is possible that regional adaptations similar to the ones previously reported^19^ may have occurred in the present study.

As suggested by the present data, ultrasound seems to represent a reliable and cheaper method alternative compared to MRI, for the estimation of changes in muscle mass with RT. The result of the present investigation seems to support the findings of two other training studies recently published by our group, in which we investigated the relationship between DXA‐derived thigh lean mass and MT.^23^, ^45^ Both studies showed good correlations between the increase in lean mass and MT just after 4 weeks of resistance training in young men^23^ and the increase/decrease in lean mass and MT after 31 days of high‐intensity interval training in an older population (males and females).^45^ However, even if considerably less expensive than MRI, and with the advantage of minimizing radiation exposure compared to CT, DXA presents some drawbacks. In fact, DXA seems to systematically underestimate the age‐related loss of lower limb lean mass compared to the loss in muscle mass assessed by MRI in older individuals.^46^ A lack of accuracy of DXA in assessing changes in lean mass with strength training compared to MRI‐derived muscle mass has also been demonstrated.^47^

When investigating muscle adaptations and metabolic aspects, DXA does not provide the possibility to measure separate muscles or muscle groups, whereas this can be easily obtained by ultrasonography. Moreover, many research facilities do not have direct access to a DXA suite and, in addition, it is still considerably more expensive than an ultrasound machine. Although DXA, compared to ultrasound, can provide more information on body composition than just the quantification of muscle mass, the aforementioned drawbacks of such a technique should be taken into account when assessing changes in muscle size.

Ultrasound‐derived MT has also been found to be a useful marker of muscle growth with RT. In fact, two previous studies demonstrated a positive correlation between MT and myofibrillar protein synthesis (expressed in terms of fractional synthetic rates) after just 3^48^ and 4 weeks^23^ of RT. This reinforces the use of ultrasound as a reliable alternative to more expensive imaging techniques for the measurement of changes in MT as an index of long‐term changes in muscle mass.

Although ultrasound has often been questioned in terms of repeatability, our group and others have demonstrated that, with appropriate operator training, measures can be highly reproducible, as shown by the ICC values from the present study (0.99) and those of previous studies (ranging between 0.997 and 0.999).^5^ It should be acknowledged that, even if ultrasound is highly reproducible, when comparing MT measurements (obtained from a single plane) to muscle volume measurements, the former might not fully explain the changes in the latter. In fact, it is known from earlier studies (ie, cross‐sectional design, which compared ultrasound‐based measurements of muscle size to MRI‐derived muscle volumes) that MT is related to VOL but explains ~80% of VOL variance.^14^, ^15^ Nevertheless, while highlighting the heterogeneity of VL hypertrophic adaptations, the present study stresses that changes in MT are locally related to the ones in ACSA following RT. This is of interest especially for studies that investigate changes in muscle size together with molecular pathways that may regulate such responses, as the muscle site of where the biopsy is taken should then be the same where MT is assessed (~50% of VL length).

Limitations of the present study were the low number and the age‐group specificity of the volunteers. However, both legs were specifically trained (unilaterally, performing different protocols) and assessed; hence, a total of 18 (ie, 9 volunteers, both legs tested and trained differently) values of VL MT, ACSA, and VOL were obtained per single time point. The advantage of training the same participants with different unilateral RT designs is that within‐subject variability in the training responses is minimized, which increases statistical power.^23^, ^49^, ^50^ This design is well established and has been adopted by several previous studies either in the knee extensors^51^, ^52^, ^53^, ^54^, ^55^ or in the elbow flexors.^50^, ^56^ Although we acknowledge that further studies are needed to specifically investigate the use of MT in different and larger populations (ie, sarcopenic and cachectic individuals, clinical settings), a good relationships between DXA‐assessed lean mass and MT measured by ultrasound in middle‐aged Japanese men and an elderly population (men and women) have been previously reported in cross‐sectional studies.^6^, ^11^ Thus, these findings seem to support the employment of ultrasound as tool for assessing skeletal muscle mass adaptations to RT by measuring MT even within an aging population.

## 5 PERSPECTIVES

The present study supports the use of ultrasound‐measured MT as a reliable tool for monitoring local long‐term hypertrophic responses (changes in VL skeletal muscle ACSA_mid_) induced by RT as an alternative to the more expensive MRI technique. However, the non‐significant correlation between the percentage changes of VL MT vs whole VOL highlights the importance of accounting for regional hypertrophy. Hence, MT should not be used to estimate changes in muscle volume. These considerations are of primary importance when assessing regional/local vs whole muscle hypertrophic adaptations, especially in relation to heterogeneous molecular/metabolic responses along the full muscle length, such as when ultrasound scan sites and muscle biopsy sites are not the same.
